# Risk Assessment and Determination of Arsenic and Heavy Metals in Fishery Products in Korea

**DOI:** 10.3390/foods12203750

**Published:** 2023-10-12

**Authors:** Do-Yeong Kim, Hyewon Jeon, Han-Seung Shin

**Affiliations:** Department of Food Science and Biotechnology, Dongguk University-Seoul, 32, Dongguk-ro, Ilsandong-gu, Goyang-si 10326, Gyeonggi-do, Republic of Korea; dykimm@dongguk.edu (D.-Y.K.); won01a8@daum.net (H.J.)

**Keywords:** fishery product, heavy metal, inductively coupled plasma mass spectrometry, direct mercury analyzer, risk assessment

## Abstract

The purpose of this study is to quantify several heavy metals (Pb, Cd, Hg, Me-Hg, and metalloid arsenic) contained in Korean fishery products (seven categories, 1186 samples) and assess their health risk. Heavy metals quantification was conducted using inductively coupled plasma mass spectrometry (ICP-MS) and a direct mercury analysis (DMA). The good linearity (R^2^ > 0.999), limits of detection (1.0–3.2 µg/kg), limits of quantification (3.1–9.6 µg/kg), accuracy (88.14–113.80%), and precision (0.07–6.02%) of the five heavy metals were obtained, and these results meet the criteria recommended by the AOAC. The average heavy metal concentrations of fishery products were in the following order: As > Cd > Pb > Hg > Me-Hg for sea algae, crustaceans, mollusks, and echinoderms, As > Hg > Me-Hg > Pb > Cd for freshwater fish and marine fish, and As > Pb > Cd > Hg > Me-Hg for tunicates. Heavy metal concentrations were lower than MFDS, EU, CODEX, and CFDA standards. In addition, the exposure, non-carcinogenic, and carcinogenic evaluation results, considering the intake of aquatic products for Koreans, were very low. It was concluded that this study will provide basic data for food safety and risk assessment.

## 1. Introduction

From a nutritional perspective, fishery products are an important resource because of their high-quality animal protein, essential amino acids, polyunsaturated fatty acids, and minerals [1,2,3]. Fish consumption is known to have a positive effect on children’s growth and brain development [4], and, in particular, omega-3 fatty acids contained in fish can prevent coronary heart disease [5]. According to the Food and Agriculture Organization of the United Nations (FAO), the global consumption of fishery products grew at an average annual rate of 3.1% from 1961 to 2017 and is expected to continue to increase. In particular, it was announced that Asia is the world’s largest seafood consumption region, consuming 24.14 kg per person per year [6]. Korea’s annual per capita consumption of seafood in 2019 was 69.8 kg [7], which is the highest consumption of seafood in Asia. Therefore, fishery products occupy a major portion and play an important role in the global and Korean diet.

Meanwhile, with rapid industrial development and various human activities, pollution of heavy metals in rivers and coastal waters is accelerating due to various kinds of living sewage and industrial wastewater. Heavy metals occur in all compartments of the marine environment and are of immense ecological importance due to their tendency to accumulate in organisms [8,9]. In particular, seaweed and bivalve mollusks can accumulate trace metals and are used as indicators of marine pollution [10]. Fish have been reported to be a major source of exposure to mercury (Hg), and the higher the intake of fish, the higher the concentration of heavy metals in the blood [11,12]. Humans can be exposed to heavy metals through ingestion of contaminated seafood, and the accumulation of heavy metals in a high concentration causes numerous human health disorders. Lead (Pb) can adversely affect the intellectual development of children and cause hypertension, nephropathy, and cardiovascular disease [13,14,15]. Chronic cadmium (Cd) exposure can induce acute toxicity in the kidney, liver, and lungs, cause nephrotoxicity, and impair immune system functions [13,16,17,18]. Arsenic (As) is associated with nervous and digestive disorders [19]. In the case of Hg, organic Hg is a fat-soluble substance that is absorbed by the digestive tract by more than 90% and has a long half-life, so it is more easily accumulated in the human body than inorganic Hg (In-Hg) [20,21]. It can also cause Minamata disease, a central nervous system disease [22]. Therefore, it is crucial to monitor the levels of heavy metals in fishery products.

A health risk assessment is defined as a systematic process used to scientifically quantify the risks associated with exposure to contamination and provides information on the correlation between exposure to heavy metals and health effects [23]. Risk assessment studies have been conducted using different food consumption models (average intake, lower and upper limits of estimated intake) in different population groups. Recently, many studies have been published on heavy metal contamination and risk assessment of fish and seafood products [24,25,26,27]. However, limited data are available concerning the potential implications of fishery products contamination to the health of local seafood consumers in Korea. The U.S. Environmental Protection Agency (USEPA) has proposed a risk assessment model to assess the carcinogenic and non-carcinogenic effects on humans exposed to heavy metals. The hazard quotient (HQ) and hazard index (HI) are used to assess non-carcinogenic risk (non-CR). HQ represents the non-CR of an individual element, and HI evaluates the total non-carcinogenic health risk for a combination of elements. The CR is estimated using the carcinogenic slope factor (CSF) related to carcinogenic metals [28]. Therefore, it is important not only to investigate the contamination levels of heavy metals in food but also to understand human exposure and health risks.

The purpose of this study was to evaluate the chemical analysis for determining the concentration of heavy metals (Pb, Cd, As, Hg, and methylmercury (Me-Hg)) contained in various fishery products in Korea. In addition, a risk assessment was conducted by calculating estimated daily intake (EDI), HQ, HI, and CR in order to evaluate the health risk of heavy metals from ingestion of aquatic products in Korea.

## 2. Materials and Methods

### 2.1. Materials

Standard solutions of Pb, Cd, and As (1000 mg/L) were purchased from Alfa Aesar (Ward Hill, MA, USA). The Hg standard (1000 mg/L) was purchased from Kanto Chemicals (Tokyo, Japan). Ultrapure nitric acid (HNO_3_, 70%) was purchased from Chemitop (Chungcheongbuk-do, Republic of Korea). Hydrogen peroxide (H_2_O_2_, 34.5%) was purchased from Samchun (Seoul, Republic of Korea). Hydrochloric acid (HCl, 30%) was purchased from Duksan (Ansan-si, Gyeonggi-do, Republic of Korea). Nylon syringe filters (25 mm, 0.45 µm) were acquired from Woongki Science Co., Ltd. (Seoul, Republic of Korea). Distilled water was purified through an Fpwps501 Ultrapure Distilled Water Purification System from Human Science Equipment Co. (Suwon-si, Gyeonggi-do, Republic of Korea).

### 2.2. Sample Collection and Preparations

**Figure 1:**
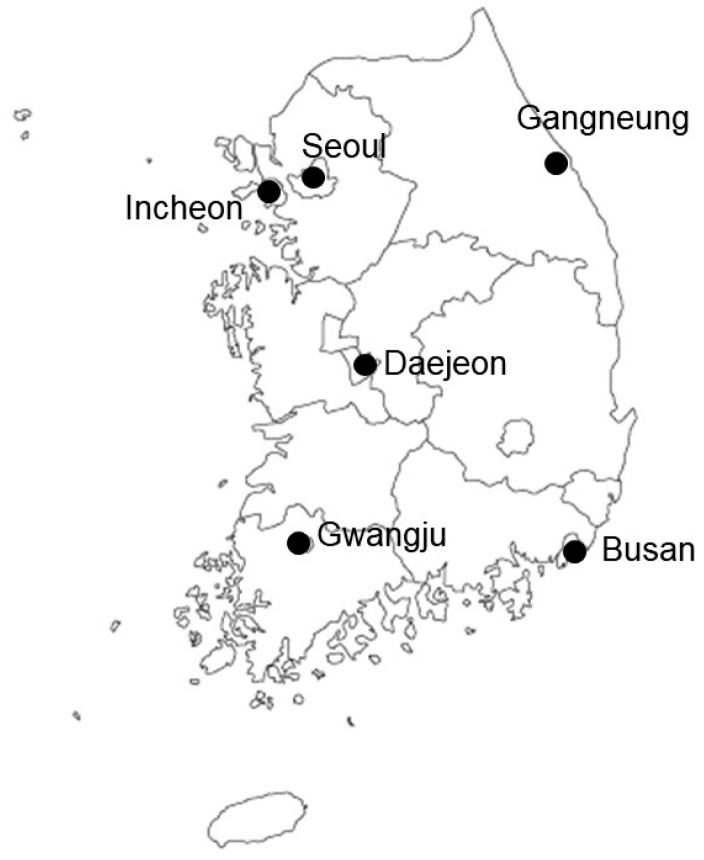
Sample collection sites in Republic of Korea.

From January to November 2021, fishery products were randomly purchased from various grocery stores and markets in six regions of the Republic of Korea (Seoul, Incheon, Daejeon, Gangneung, Busan, and Gwangju) with high population density and high fishery product consumption (Figure 1). After removing the non-edible part of each product, the food sample was homogenized with a blender and stored in a conical tube at −18 °C. The samples were sorted into seven categories (Table 1). The first group was sea algae (*n* = 105), including laver, kelp, sea mustard, and hijiki. The second group was freshwater fish (*n* = 87), including catfish, carp, mudfish, and mandarin fish. The third group was marine fish (*n* = 559), including mackerel, cutlassfish, croaker, and shark. The fourth group was crustaceans (*n* = 65), including shrimp, crab, lobster, and king crab. The fifth group was mollusks (*n* = 320), including mussel, oyster, cockle, and abalone. The sixth group was tunicates (*n* = 30), including sea squirt and warty sea squirt. The seventh group was echinoderms (*n* = 20), including sea urchin and sea cucumber.

### 2.3. Sample Preprocessing

Sample preprocessing for a Pb, Cd, As, and Me-Hg analysis was carried out as previously reported with minor modifications [29].

#### 2.3.1. Pb, Cd, and As Analysis

Samples were acid-digested using a microwave digestion system (ETOS 10, Milestone, Bergamo, Italy). Approximately 0.5 g of the sample was placed into a Teflon vessel, and then 7 mL of ultrapure HNO_3_ was added to the sample. The microwave digestion process was as follows: heating for 15 min to 65 °C and isothermal for 5 min, heating for 10 min to 180 °C and isothermal for 30 min, then cooling to 40 °C for 30 min. After digestion, the solution was diluted with ultrapure distilled water to 50 mL and filtered through a 0.45 µm nylon syringe filter for an inductively coupled plasma mass spectrometry (ICP-MS) analysis.

#### 2.3.2. Me-Hg Analysis

Approximately 1.0 g of the sample was placed into a 50 mL plastic centrifuge tube, followed by the addition of 15 mL of toluene (99.5%), 10 mL of sodium chloride (NaCl, 25%), and 0.5 mL of HCl (98%). Extraction was performed for 2 min using a shaker (RS-1, JEIO TECH, Daejeon, Republic of Korea), and the extract was centrifuged at 2500 rpm for 20 min. The toluene supernatant was aliquoted, and 5 mL of an L-cysteine solution was added, followed by shaking for 5 min. The L-cysteine layer was used as the final solution, and 0.1 g of the solution was placed into a quartz sample boat for an analysis with a direct mercury analyzer (DMA).

### 2.4. Instrument Optimization for ICP-MS Analysis

ICP-MS analysis was executed as mentioned previously with minor modifications [29]. Heavy metals quantification was carried out with ICP-MS (iCAP-RQ, Thermo Fisher Scientific, Waltham, MA, USA). The ICP-MS was optimized daily with a tuning solution (iCAP Q/Qnova Tune Solution, Thermo Fisher Scientific, Waltham, MA, USA) to maximize the signal and minimize the interference effect of polyatomic ions and doubly charged ions. This was achieved by adjusting the torch position, nebulizer gas flow, and make-up gas flow. The optimized parameters are as follows: analysis mode—KED; argon (Ar) gas (high-purity, 99.99%) flow rates of auxiliary gas flow rate—0.8 mL/min, nebulizer gas flow rate—1.0 mL/min, and helium (He) gas (high-purity, 99.99%) flow rate—5.3 mL/min; lens voltage—6.25 V; radio frequency (RF) power—1500 W; and analyte isotopes ^208^Pb, ^111^Cd, and ^75^As.

### 2.5. Instrument Optimization for DMA Analysis

The DMA analysis was performed as previously described with minor modifications [29]. Hg and Me-Hg were measured with the heat vaporization gold amalgam method using a DMA-80 evo (Milestone, Sorisole, Italy). The quartz sample boat (DMA 8347, Milestone, Sorisole) used for the analysis was washed with distilled water using a sonication system, the height of the empty boat was set to lower than 0.0030, and the sample analysis was performed. For the analysis of Hg, about 0.03–0.04 g of the homogenized sample was taken without additional pretreatment. The Me-Hg analysis was performed by taking 1 µL of the pretreated sample. The optimized parameters are as follows: drying temperature—650 °C; drying time—90 s; decomposition temperature—650 °C; decomposition time—180 s; purge time—60 s; amalgamator heating temperature—850 °C; amalgam heating time—12 s; and recording time—30 s.

### 2.6. Method Validation for Quality Assurance of Analysis

The ICP-MS and DMA analysis methods were validated for linearity (R^2^), detection limit (LOD), quantitation limit (LOQ), precision (%), and accuracy (%). Mackerel was selected as a representative matrix of aquatic products to validate the analysis methods. To evaluate the linearity, calibration curves were constructed by analyzing five calibration standards of each heavy metal. The linear relationship between the concentration of heavy metals and the responses of the analyte is expressed as the square of the correlation coefficient (R^2^). Precision and accuracy were tested by spiking three concentrations of heavy metal standards into the sample. Both inter-day and intra-day precision and accuracy were determined. Inter-day was analyzed for 3 different days, and the intra-day analysis was examined three times on a single day. The concentration of heavy metals was calculated using a calibration curve. The precision was confirmed through the relative standard deviation (RSD%). The accuracy was obtained by calculating the recovery rate using Equation (1):

Recovery (%) = (*C*_f_ − *C*_u_)/*C*_a_ (1)

where *C*_f_ is the concentration of the spiked sample, *C*_u_ is the concentration of the sample, and *C*_a_ is the concentration of the standard solution.

### 2.7. Health Risk Assessment

Human health risks were estimated as previously reported [30]. To assess the human health risks of heavy metals in fishery products in Korea, the EDI of the metals according to the intake of aquatic products was calculated, and the non-CR and the CR probability were evaluated, respectively, using the formulas for EDI (Equation (2)), HQ (Equation (3)), HI (Equation (4)), and CR (Equation (5)), as described below:

EDI (µg/kg/day) = (C × IR/BW)(2)

where C is the concentration of heavy metals (mg/kg), IR is the intake rate (g/day), and BW is the average body weight (kg).

IR was based on the data from the 2020 Korea National Health and Nutrition Examination Survey (KNHANES) provided by the Korea Disease Control and Prevention Agency [31]. The average weight of a Korean adult is 65.55 kg, which was obtained from the health checkup statistical data of the National Health Insurance Service [32].

#### 2.7.1. Non-CR

The non-CR was assessed using the HQ, defined as the ratio of the EDI (µg/kg/day) to the oral reference dose (RfD, µg/kg/day) for metal elements. The RfD values for Pb, Cd, As, Hg, and Me-Hg were 3.5, 1.0, 0.3, 0.3, and 0.4 μg/kg/day, respectively [33,34].

HQ = EDI/RfD(3)

HI = HQ (Pb) + HQ (Cd) + HQ (As) + HQ (Hg) (4)

where HI is expressed as the sum of the HQs for all trace elements.

#### 2.7.2. CR

The CR was calculated using Equation (5):

CR = EDI × CSF/1000(5)

where CSF is oral CSF (mg/kg/day).

CSF values were established only for In-As and Pb. The CSF for In-As and Pb was 1.5 and 0.0085 mg/kg/day, respectively [30].

### 2.8. Statistical Analysis

All analyses were executed in triplicate, and the data are expressed as the mean ± standard deviation (s.d.). All statistical analyses were executed by using SPSS 21.0 software (IBM, Chicago, IL, USA) and a one-way analysis of variance (ANOVA) was conducted.

## 3. Results and Discussion

### 3.1. Method Validation for Heavy Metals Analysis

To evaluate the linearity, calibration curves were constructed by analyzing five calibration standards of each heavy metal with concentrations of 0.25, 0.5, 1.0, 5.0, and 10.0 µg/kg for Pb, Cd, and As and 0.5, 1.0, 5.0, 10, and 20 µg/kg for Hg and Me-Hg. The calibration curves were found to be linear in the concentration range tested, and the R^2^ values were higher than 0.999 for all elements. For the mackerel matrix, the LOD ranged from 1.0 to 3.2 µg/kg, and the LOQ ranged from 3.1 to 9.6 µg/kg. The linearity, LOD, and LOQ of the heavy metals are presented in Table 2. The good linearity (R^2^ > 0.999) of our method validation is obtained. The LOD and LOQ of all elements were higher than those of home meal replacement (HMR) [29] and vegan food [35] and similar to those of shrimp and shellfish [36]. For HMR, vegan food, and shrimp and shellfish, the LOD ranges were 0.021–2.81, 0.001–0.185, and 1.2–2.8 µg/kg, respectively, and the LOQ ranges were 0.05–8.51, 0.07–0.616, and 3.7–8.4 µg/kg, respectively. This observation is due to the effect of mass interference of polyatomic ions generated with the combination of Ar, a gas supplied to the ICP-MS apparatus, and chlorine ions (Cl) in salt (NaCl). There is also the effect of ionization inhibition due to the high salt content of fishery products [37].

The accuracy (%) and precision (coefficient of variation (CV), %) of intra-day and inter-day results of a standard mixture of five heavy metals at three concentrations (low, middle, and high) are presented in Table 3. The accuracy and precision of five heavy metals in the mackerel matrix were in the ranges of 88.14–113.00% and 0.07–2.51% (intra-day), and 97.4–113.8% and 0.11–6.02% (inter-day), respectively. These results satisfied the criteria of accuracy (70–125%) and precision (<15%) recommended by the Association of Official Analytical Chemists (AOAC) [38]. The results provided in Table 2 and Table 3 satisfied all the linearity, precision, and accuracy standards required by the AOAC. Therefore, the validated analytical method is sensitive enough and suitable for the quantification of five heavy metals in fishery products.

### 3.2. Heavy Metal Contents in Fishery Products

The concentrations of heavy metals were analyzed in 1186 samples of fishery products (Table 4). All samples were repeated in triplicate. Me-Hg was analyzed only in samples in which Hg was detected.

Our research results satisfied all of the heavy metal standards suggested by the Ministry of Food and Drug Safety in Korea (MFDS) [39]. When compared with the standards presented by the European Union (EU) [40], the Codex Alimentarius Commission (CODEX) [41], and the China Food & Drug Administration (CFDA) [42], the average contents of each category of fishery products in our study satisfied the limit. Unlike Korea, the EU, and the CODEX, there is a Pb standard (0.5 mg/kg) for sea algae in China, and the maximum Pb content (0.109 mg/kg) of sea algae in our study did not exceed this standard. In the EU, the Cd standard (0.05 mg/kg) for fish is lower than in Korea (0.2 mg/kg) and China (0.1 mg/kg). The maximum concentration of Cd in marine fish was 0.098 mg/kg, which exceeds the EU limit. The EU sets a standard for Hg (1.0 mg/kg) in deep seawater and predatory fish, but there is no standard for Me-Hg. The maximum content of Hg (2.245 mg/kg) found in marine fish in this study exceeds the EU standard by more than twice. However, the maximum content of Me-Hg, which is known to be the most toxic of Hg species, is 0.084 mg/kg according to EU standards. It is very low compared to standards presented by Korea (1.0 mg/kg), the CODEX (1.7 mg/kg), and China (1.0 mg/kg).

The mean heavy metal concentrations of the seven categories were in the following order: As > Cd > Pb > Hg > Me-Hg for sea algae, crustaceans, mollusks, and echinoderms, As > Hg > Me-Hg > Pb > Cd for freshwater fish and marine fish, and As > Pb > Cd > Hg > Me-Hg for tunicates. Except for freshwater fish and marine fish, the content distribution of heavy metals in the remaining five categories of fishery products showed a similar trend; As, Cd, and Pb contents were high, and the Hg content was relatively low. In freshwater and marine fish, the As and Hg contents were dominant, while the Pb and Cd contents ranked relatively low.

The heavy metal As was the highest in all seafood categories. In studies analyzing the As content of HMR [29] and agricultural products [43], the As contents were 0.009–0.048 and 0.004–0.103 mg/kg, respectively, whereas our study showed a higher As content of 0.151–3.626 mg/kg in aquatic products. The heavy metal As occurs naturally in the Earth’s crust, is easily released into natural waters, undergoes chemical changes in the aquatic environment, and eventually flows into the ocean and becomes concentrated [44]. This is expected because of the high accumulation of more As in aquatic products than in other food types. In addition, another study reported that sea shrimp had a higher total As content than freshwater shrimp and that the predominant As species in sea shrimp and sea salt were consistent [45]. This corroborates with our study results, which showed that marine fish had an 11 times higher total As content than freshwater fish. These studies may support the result of high As content in sea creatures.

Hg and Me-Hg were particularly high in fish and accounted for the highest portion after As in fish. In the marine environment, Hg is mostly present as In-Hg. In-Hg is converted to Me-Hg, an organic form, with the activity of microorganisms in soils and sediments [46]. Me-Hg is demethylated with a photochemical reaction and converted to In-Hg. However, in the deep sea, where sunlight does not reach, the possibility of photochemical reactions is low, and the deeper the water, there exists high concentrations of Me-Hg [47]. In addition, Me-Hg is found in a large amount in predatory fish species because of bioaccumulation as they move up the food chain [48]. Therefore, in the results of this study, it is judged that the Hg and Me-Hg contents are high in fish, especially in marine fish, including deep-sea fish and predatory fish. Hg and Me-Hg contents ranging from 0.0102 to 0.108 and 0.001 to 0.107 mg/kg, respectively, in marine fish from the Caspian Sea [49]; 0.0107 to 0.049 and 0.017 to 0.300 mg/kg, respectively, in marine fish from the Persian Gulf [49]; and <LOD to 3.51 and <LOD to 1.12 mg/kg, respectively, in commercially important fishes in Japan [50] have been reported. When compared with Hg (0.003–2.245 mg/kg) and Me-Hg (LOQ-0.322 mg/kg) in marine fish in this study, Hg was higher than that found in marine fish from the Caspian Sea and Persian Gulf and lower than that found in commercially important fishes in Japan. Me-Hg was higher than that in marine fish from the Caspian Sea, similar to that in marine fish from the Persian Gulf, and lower than that found in commercially important fishes in Japan.

Cd had a notably high content in mollusks and crustaceans. The mollusk samples in this study included shellfish and cephalopods. Shellfish are sessile, have a relatively long lifespan, and accumulate pollutants in the body, so they are greatly affected by the habitat environment. Shellfish ingest and accumulate heavy metals through adsorption and absorption through the body surface, absorption of suspended matter in seawater through filter feeding, and intake of plankton. In addition, studies have reported that Cd is particularly concentrated in the liver and kidney [51,52,53]. Cephalopods are characterized by a high content of heavy metals because they feed on crustaceans, shellfish, and various mollusks, which contain many benthic organisms [54,55]. In addition, Cd was accumulated to a higher extent in the lower-layer sedentary organisms than in the surface migratory organisms [56]. Due to these habitat and dietary characteristics of mollusks and crustaceans, they are judged to have a higher Cd content than other seafood categories. The average content of Cd in this study was <LOQ-0.563 mg/kg, lower than in China’s Sanmen Bay (0.18–9.64 mg/kg) [57] and similar to Turkey’s Aegean Sea coast (0.04–0.52 mg/kg) [58]. Although the content of Cd was the highest in mollusks, it is not a level to be concerned about when compared with foreign studies.

In Spain, 18 algae food products were analyzed, and the concentrations of Pb, Cd, As, and Hg were <0.05–1.33, 0.03–1.9, 2.3–141, and 0.004–0.04 mg/kg, respectively [59]. Compared with our research in marine algae, the Hg content was similar, but the Pb, Cd, and As levels were lower. In both studies, the content of each element in sea algae showed a similar trend of As > Cd > Pb > Hg. In studies conducted in Bosnia and Herzegovina, the average concentrations of Pb, Cd, As, and Hg were 0.008, 0.014, 0.960, and 0.743 mg/kg in marine fish and 0.006, 0.003, 0.070, and 0.063 mg/kg in freshwater fish, respectively [60]. By comparison, in our study, marine fish showed higher levels of Pb and As, similar levels of Cd, and significantly low Hg. In the case of freshwater fish, Pb and As were higher, and Hg and Cd were comparable with our current study. The concentration ranges for Pb, Cd, As, and Hg in fish, crustaceans, and echinoderms from the Tuscany Coast (northern Italy) were <LOQ-0.03, <LOQ-0.01, 0.42–28.7, and 0.04–1.52 mg/kg in fish; 0.03–0.33, 0.06–0.35, 1.51–35.80, and 0.03–0.33 mg/kg in crustaceans; and 0.52–0.85, 0.01–0.17, 2.84–3.74, and 0.04–0.041 mg/kg in echinoderms, respectively [61]. These results represent a significantly higher range than found in our study, which is because the samples were taken from an industrially active area of the Tuscany Coast. This suggests that industrial activity has a large impact on the contamination of fishery products with heavy metals and indicates that Korean aquatic products are less contaminated with heavy metals compared to some other countries. The content of Pb, Cd, As, and Hg in marine fish from the Black Sea was reported to be 0.03–0.08, 0.005–0.015, 0.38–1.10, and 0.05–0.16 mg/kg, respectively [62]. Results in our study showed that the heavy metals content was overall higher, especially As and Hg. It is probably because our sample contains deep-water and predatory fish species known to contain high levels of Hg [63,64]. A study of the contents of heavy metals in mollusks collected from the Atlantic Coast reported ranges from 0.160 to 0.410 mg/kg for Pb, 0.045 to 0.148 mg/kg for Cd, 1.611 to 13.497 mg/kg for As, and 0.006 to 0.046 mg/kg for Hg [65]. In mollusks collected from the Bohai Coast, the Pb, Cd, As, and Hg concentrations were reported as 0.10–0.22, 0.22–1.3, 1.16–3.08, and 0.006–0.010 mg/kg, respectively [66]. Our results for Pb and Cd were similar and higher, respectively, to those reported from the Atlantic Coast but were lower than those from the Bohai Coast. As and Hg were the highest in our study.

### 3.3. Health Risk Assessment

#### 3.3.1. Exposure Assessment

The EDI of five heavy metals in fishery products was calculated by considering the BW of Koreans for the average daily intake amount and heavy metals contamination for each item. The results are presented in Table 5. The highest EDI values for Pb, Cd, and As all belonged to crustaceans: 5.84 × 10^−4^, 2.56 × 10^−3^, and 4.23 × 10^−2^ µg/kg/day, respectively. Hg and Me-Hg had the highest EDI values in marine fish: 1.57 × 10^−3^ and 2.68 × 10^−4^, respectively. The Joint FAO/WHO Expert Committee on Food Additives (JECFA) set the provisional tolerance weekly intake (PTWI) of Hg and Me-Hg to 4.0 and 1.6 µg/kg/week, respectively, and for Cd with a relatively long half-life, set the provisional tolerance monthly intake (PTMI) to 25 µg/kg/month [67]. The average estimated weekly intake (EWI) of Hg and Me-Hg in fishery products was 2.16 × 10^−3^ and 4.17 × 10^−4^ µg/kg/week, respectively, which were 0.05% and 0.0003% of the PTWI set by the JECFA, respectively. The estimated monthly intake of Cd compared to the PTMI set by the JECFA was 0.08%. Therefore, exposure to heavy metals through fishery products is considered safe in Korea.

#### 3.3.2. Non-CR

The assessment of chronic health risks of heavy metals from consumption of fishery products was determined using HQ, which is the ratio of heavy metal exposure to oral RfD. If the HQ is <1, the non-carcinogenic health risk is judged to be negligible [68]. Because health risk is the accumulation of multiple elements, the total health risk of heavy metals was evaluated using HI, which is the sum of the HQs for each element. Me-Hg is included in Hg, and so to avoid duplication of the health risk, the HQ of Me-Hg was excluded and added up.

The values for HQ and HI are presented in Table 6. Pb, Cd, and As had the highest HQ value in crustaceans, while Hg and Me-Hg were the highest in marine fish. It has the same trend as the heavy metal content and exposure assessment. Because all HQ values were <1, the possibility of non-CR is very low. The average HQ of each metal can be ranked in the following order: As (5.07 × 10^−2^) > Hg (1.03 × 10^−3^) > Cd (6.74 × 10^−4^) > Me-Hg (1.49 × 10^−4^) > Pb (6.79 × 10^−5^). Conversely, in the exposure assessment, the average EDI of each metal decreased in the following order: As > Cd > Pb > Hg > Me-Hg. This difference is thought to be due to the low RfD of Hg and the high RfD of Pb. The HI values in fishery products ranged from 1.36 × 10^−3^ in freshwater fish to 1.45 × 10^−1^ in crustaceans. The HI values of the fishery products were <1, demonstrating that the non-CR from ingestion of these samples is not significant to human health. On the other hand, according to previous research on human risk assessment of toxic elements (As, Cd, Hg, and Pb) in marine fish from the Amazon Coast, HQ values for As exhibited as above 1 in 25 of the 27 commercial species samples, while HQ values of Cd were less than 1 in all samples [27]. This indicates that different seafood organisms may have differences in heavy metal contamination and health risks depending on their habitat.

#### 3.3.3. CR

CR is determined based on the carcinogenic potential of the heavy metals and information on exposure to the substance. The CR is calculated by multiplying the CSF by EDI [69], which is established only for In-As and Pb by the USEPA. The most toxic of the As species is In-As [70]. In order to protect Korean consumers, in this study, As was assumed to be In-As, and the CR was calculated. The CR values are shown in Table 7. In general, a CR value in the range of 10^−4^ to 10^−6^ is considered appropriate to protect human health. However, considering the unknown exposure, if the estimated CR value is 10^−6^ or less, it is considered a reasonable safe range [64]. All CR values of Pb in aquatic products were below 1 × 10^−6^, which means that the potential cancer risk from Pb is negligible. In the case of As, all types of fishery products except freshwater fish showed CR values between 1 × 10^−4^ and 1 × 10^−6^, meaning the potential CR caused by As is at an acceptable level. In addition, because the CR value was calculated based on In-As with a high CR, the CR from total As is thought to be safe.

## 4. Conclusions

In this study, the concentration of five heavy metals (Pb, Cd, As, Hg, and Me-Hg) in Korean fishery products was investigated, and the human risk of heavy metals caused by ingestion of aquatic products was evaluated. The analytical method used in this study satisfied the condition suggested by the CODEX, and it has been verified that it is a reliable method for measuring the content of heavy metals. The concentration of heavy metals was lower than the MFDS, EU, CODEX, and CFDA standards, and the exposure, non-carcinogenic, and carcinogenic evaluation results, considering the intake of aquatic products for Koreans, were very low. Therefore, it was concluded that the current level of contamination of heavy metals in Korean fishery products is safe. This study will provide important insights concerning potential human health impacts as a result of the consumption of potentially hazardous species of fishery products in Korea. In addition, this study will provide basic data for food safety assessment and risk management. However, further study is needed to understand the relationship between contaminant levels and ecological species aspects (body size, habitat, etc.) and seasonal variations.

## Figures and Tables

**Table 1:** Name (English and scientific name) and number of samples belonging to seven categories.

No.	Category	Name		Number
		Common	Scientific	
1	Sea algae (*n* = 105)	Laver	*Porphyra purpurea*	15
		Kelp	*Saccharina japonica*	15
		Seaweed fulvescens	*Capsosiphon fulvescens*	15
		Gulfweed	*Sargassum fulvellum*	15
		Sea mustard	*Undaria pinnatifida*	15
		Hijiki	*Hizikia fusiforme*	15
		Green laver	*Enteromorpha*	15
2	Freshwater fish (*n* = 87)	Catfish	*Silurus asotus*	14
		Carp	*Cyprinus carpio*	14
		Mudfish	*Misgurnus mizolepis*	15
		Mandarin fish	*Siniperca scherzeri*	14
		Cherry salmon	*Oncorhynchus masou*	15
		Salmon	*Oncorhynchus keta*	15
3	Marine fish (*n* = 559)	Ray	Batoidea	30
		Cutlassfish	*Trichiurus lepturus*	32
		Mackerel	*Scomber japonicus*	30
		Striped marlin	*Tetrapturus audax*	15
		Swordfish	*Xiphias gladius*	15
		Pacific saury	*Cololabis saira*	32
		Flatfish	*Paralichthys olivaceus*	25
		Patagonian toothfish	*Dissostichus eleginoides*	20
		Bigeye tuna	*Thunnus obesus*	25
		Sailfin sandfish	*Arctoscopus japonicus*	30
		Black rock fish	*Sebastes inermis*	20
		Anchovy	*Engraulis japonicus*	35
		Pollack	*Theragra chalcogramma*	30
		Japanese icefish	*Salangichthys microdon*	30
		Blowfish	Tetraodontiformes	35
		Croaker	*Larimichthys polyactis*	35
		Bluefin tuna	*Thunnus thynnus*	30
		Pacific herring	*Clupea pallasii*	30
		Sciaenoid fish	*Miichthys miiuy*	30
		Shark	*Carcharodon carcharias*	30
4	Crustaceans (*n* = 65)	Shrimp	Caridea	20
		Crab	Brachyura	15
		Lobster	Nephropidae	15
		King crab	Paralithodes	15
5	Mollusks (*n* = 320)	Squid	*Todarodes pacificus*	25
		Beka squid	*Loliolus beka*	25
		Octopus	*Enteroctopus dofleini*	25
		Webfoot octopus	*Amphioctopus fangsiao*	25
		Small octopus	*Octopus minor*	25
		Mussel	*Mytilus unguiculatus*	25
		Oyster	*Crassostrea gigas*	25
		Cockle	*Tegillarca granosa*	25
		Abalone	*Haliotis discus*	25
		Razor clam	*Solen strictus*	25
		Short-neck clam	*Ruditapes philippinarum*	25
		Ark shell	*Scapharca broughtonii*	25
		Fat innkeeper worm	*Urechis unicinctus*	20
6	Tunicates (*n* = 30)	Sea squirt	*Halocynthia roretzi*	15
		Warty sea squirt	*Styela clava*	15
7	Echinoderms (*n* = 20)	Sea urchin	Echinoidea	10
		Sea cucumber	Holothuroidea	10

**Table 2:** The LOD, LOQ, linearity equation, and R^2^ of the five heavy metals.

Heavy Metals	Food Matrix			
	Mackerel			
	LOD ^a^ (mg/kg)	LOQ ^b^ (mg/kg)	Linearity Equation	R^2^
Pb	0.001	0.004	y = 129850x + 4690.1	1.0000
Cd	0.001	0.004	y = 2585x + 263.64	1.0000
As	0.002	0.005	y = 2585x + 263.64	0.9995
Hg	0.001	0.003	y = −0.0007x^2^ + 0.051x − 0.0019	0.9998
Me-Hg	0.003	0.010	y = −0.0007x^2^ + 0.0556x − 0.0025	0.9999

^a^ Limit of detection (LOD) = 3.3 × σ/S; σ is the standard deviation of the response, and S is the slope of the calibration curve. ^b^ Limit of quantification (LOQ) = 10 × σ/S.

**Table 3:** Accuracy and precision of the five heavy metals.

Heavy Metals	Accuracy (%)						Precision (%)					
	Intra-Day (*n* = 3)			Inter-Day (*n* = 3)			Intra-Day (*n* = 3)			Inter-Day (*n* = 3)		
	Low	Middle	High	Low	Middle	High	Low	Middle	High	Low	Middle	High
Pb	106.40	88.24	95.90	108.70	111.30	99.90	2.30	2.06	0.29	5.17	6.42	1.83
Cd	95.60	93.50	100.10	97.40	100.10	99.90	0.52	1.48	0.07	6.84	5.13	0.11
As	113.00	102.04	91.720	104.53	108.31	102.29	2.51	0.85	0.53	5.95	6.02	5.17
Hg	108.71	104.01	110.29	113.80	104.86	110.52	2.51	1.55	0.61	3.06	2.42	0.91
Me-Hg	102.90	103.81	97.37	103.30	103.86	97.6	0.72	0.44	0.11	0.94	0.43	0.28

**Table 4:** Mean, max, and min concentration of heavy metals in fishery products.

Category	Heavy Metals (mg/kg)														
	Pb			Cd			As			Hg			Me-Hg		
	Mean	Max	Min	Mean	Max	Min	Mean	Max	Min	Mean	Max	Min	Mean	Max	Min
Sea algae	0.048 (0.052)	0.109	0.0178	0.079 (0.085)	0.156	0.012	3.626 (3.921)	10.063	0.397	0.002 (0.01)	0.012	ND	ND	ND	ND
Freshwater fish	0.012 (0.018)	0.03	0.003	0.003 (0.01)	0.018	ND	0.151 (0.26)	0.398	0.037	0.066 (0.108)	0.236	0.004	0.023 (0.066)	0.084	ND
Marine fish	0.014 (0.03)	0.047	0.002	0.013 (0.027)	0.098	ND	1.776 (2.828)	10.258	0.232	0.205 (0.639)	2.245	0.003	0.035 (0.111)	0.322	ND
Crustaceans	0.041 (0.143)	0.161	0.008	0.18 (0.505)	0.408	0.0003	2.969 (3.143)	4.754	0.252	0.021 (0.035)	0.049	0.005	0.004 (0.008)	0.007	ND
Mollusks	0.067 (0.114)	0.2653	0.004	0.202 (0.334)	0.563	ND	3.272 (7.827)	21.919	0.024	0.021 (0.056)	0.227	0.001	0.007 (0.035)	0.074	ND
Tunicates	0.047 (0.052)	0.09	0.03	0.015 (0.01)	0.02	0.019	0.311 (0.278)	0.513	0.347	0.0003 (0.001)	0.001	0.001	ND	ND	ND
Echinoderms	0.009 (0.009)	0.01	0.008	0.022 (0.05)	0.037	0.006	0.782 (0.84)	1.118	0.445	0.0002 (0.001)	0.0004	ND	ND	ND	ND

ND, not detected, the lower limit of detection. Standard deviations in parentheses. The maximum and minimum values for each category represent the maximum and minimum averages of the analysis items belonging to the category.

**Table 5:** Estimated exposure assessment of five heavy metals for fishery products.

Category	Heavy Metal							
	Pb	Cd		As	Hg		Me-Hg	
	EDI (µg/kg/day)	EDI (µg/kg/day)	EMI (µg/kg/month)	EDI (µg/kg/day)	EDI (µg/kg/day)	EWI (µg/kg/week)	EDI (µg/kg/day)	EWI (µg/kg/week)
Sea algae	3.27 × 10^−4^	5.39 × 10^−4^	1.62 × 10^−2^	2.47 × 10^−2^	1.36 × 10^−5^	9.55 × 10^−5^	-	-
Freshwater fish	2.24 × 10^−5^	5.59 × 10^−6^	1.68 × 10^−4^	2.81 × 10^−4^	1.23 × 10^−4^	8.61 × 10^−4^	4.29 × 10^−5^	3.00 × 10^−4^
Marine fish	1.07 × 10^−4^	9.96 × 10^−5^	2.99 × 10^−3^	1.36 × 10^−2^	1.57 × 10^−3^	1.10 × 10^−2^	2.68 × 10^−4^	1.88 × 10^−3^
Crustaceans	5.84 × 10^−4^	2.56 × 10^−3^	7.69 × 10^−2^	4.23 × 10^−2^	2.99 × 10^−4^	2.09 × 10^−3^	5.70 × 10^−5^	3.99 × 10^−4^
Mollusks	4.71 × 10^−4^	1.42 × 10^−3^	4.26 × 10^−2^	2.30 × 10^−2^	1.48 × 10^−4^	1.03 × 10^−3^	4.92 × 10^−5^	3.45 × 10^−4^
Tunicates	1.31 × 10^−4^	4.17 × 10^−5^	1.25 × 10^−3^	8.65 × 10^−4^	8.34 × 10^−7^	5.84 × 10^−6^	-	-
Echinoderms	2.03 × 10^−5^	4.96 × 10^−5^	1.49 × 10^−3^	1.76 × 10^−3^	4.51 × 10^−7^	3.16 × 10^−6^	-	-

EDI, estimated daily intake; EMI, estimated monthly intake; EWI, estimated weekly intake.

**Table 6:** Hazard quotient (HQ) and hazard index (HI) for heavy metals in fishery products.

Category	HQ					HI
	Pb	Cd	As	Hg	Me-Hg	Total
Sea algae	9.36 × 10^−5^	1.80 × 10^−4^	8.25 × 10^−2^	4.55 × 10^−5^	0.00 × 10^0^	8.28 × 10^−2^
Freshwater fish	6.39 × 10^−6^	1.86 × 10^−6^	9.38 × 10^−4^	4.10 × 10^−4^	1.07 × 10^−4^	1.36 × 10^−3^
Marine fish	3.07 × 10^−5^	3.32 × 10^−5^	4.54 × 10^−2^	5.24 × 10^−3^	6.71 × 10^−4^	5.07 × 10^−2^
Crustaceans	1.67 × 10^−4^	8.55 × 10^−4^	1.41 × 10^−1^	9.97 × 10^−4^	1.42 × 10^−4^	1.43 × 10^−1^
Mollusks	1.35 × 10^−4^	4.74 × 10^−4^	7.67 × 10^−2^	4.92 × 10^−4^	1.23 × 10^−4^	7.78 × 10^−2^
Tunicates	3.73 × 10^−5^	1.39 × 10^−5^	2.88 × 10^−3^	2.78 × 10^−6^	0.00 × 10^0^	2.94 × 10^−3^
Echinoderms	5.80 × 10^−6^	1.65 × 10^−5^	5.88 × 10^−3^	1.50 × 10^−6^	0.00 × 10^0^	5.90 × 10^−3^

**Table 7:** Estimated carcinogenic risk of Pb and As for fishery products.

Category	CR	
	Pb	As
Sea algae	2.78 × 10^−9^	3.71 × 10^−5^
Freshwater fish	1.90 × 10^−10^	4.22 × 10^−7^
Marine fish	9.12 × 10^−10^	2.04 × 10^−5^
Crustaceans	4.96 × 10^−9^	6.34 × 10^−5^
Mollusks	4.00 × 10^−9^	3.45 × 10^−5^
Tunicates	1.11 × 10^−9^	1.30 × 10^−6^
Echinoderms	1.72 × 10^−10^	2.64 × 10^−6^

## Data Availability

The data used to support the findings of this study can be made available by the corresponding author upon request.

## References

[1] Channing D., Young G. (1953). 503. Amino-acids and peptides. Part X. The nitrogenous constituents of some marine algae. J. Chem. Soc..

[2] Robledo D., Freile Pelegrín Y. (1997). Chemical and mineral composition of six potentially edible seaweed species of Yucatan. Bot. Mar..

[3] Sargent J., Bell G., McEvoy L., Tocher D., Estevez A. (1999). Recent developments in the essential fatty acid nutrition of fish. Aquaculture.

[4] Zupanc G. (2006). Neurogenesis and neuronal regeneration in the adult fish brain. J. Comp. Physiol. A.

[5] Kromhout D., Bosschieter E.B., Coulander C.d.L. (1985). The inverse relation between fish consumption and 20-year mortality from coronary heart disease. N. Engl. J. Med..

[6] Food and Agriculture Organization of the United Nations (FAO) (2020). The State of World Fisheries and Aquaculture (SOFIA).

[7] Korea Rural Economic Institute (KREI) (2019). 2019 Food Balance Sheet.

[8] Amiard J., Amiard-Triquet C., Berthet B., Metayer C. (1987). Comparative study of the patterns of bioaccumulation of essential (Cu, Zn) and non-essential (Cd, Pb) trace metals in various estuarine and coastal organisms. J. Exp. Mar. Biol..

[9] Stoeppler M. (1992). Hazardous Metals in the Environment.

[10] Bryan G., Langston W. (1992). Bioavailability, accumulation and effects of heavy metals in sediments with special reference to United Kingdom estuaries: A review. Environ. Pollut..

[11] Kim Y.A., Kim Y.-N., Cho K.-D., Kim M.Y., Kim E.J., Baek O.-H., Lee B.-H. (2011). Blood heavy metal concentrations of Korean adults by seafood consumption frequency: Using the fourth Korea National Health and Nutrition Examination Survey (KNHANES IV), 2008. Korean J. Nut.

[12] Rahbar M.H., Samms-Vaughan M., Loveland K.A., Ardjomand-Hessabi M., Chen Z., Bressler J., Shakespeare-Pellington S., Grove M.L., Bloom K., Pearson D.A. (2013). Seafood consumption and blood mercury concentrations in Jamaican children with and without autism spectrum disorders. Neurotox. Res..

[13] Blakley B. (1985). The effect of cadmium chloride on the immune response in mice. Can. J. Comp. Med..

[14] Voors A.W., Johnson W.D., Shuman M.S. (1982). Additive statistical effects of cadmium and lead on heart related disease in a North Carolina autopsy series. Arch. Environ. Health.

[15] Wasserman G.A., Liu X., Lolacono N.J., Factor-Litvak P., Kline J.K., Popovac D., Morina N., Musabegovic A., Vrenezi N., Capuni-Paracka S. (1997). Lead exposure and intelligence in 7-year-old children: The Yugoslavia Prospective Study. Environ. Health Perspect..

[16] Elinder C.-G., Kjellström T., Friberg L., Linnman B.L.L. (1976). Cadmium in kidney cortex, liver, and pancreas from Swedish autopsies. Arch. Environ. Health.

[17] Friberg L. (1984). Cadmium and the kidney. Environ. Health Perspect..

[18] Waalkes M.P. (2000). Cadmium carcinogenesis in review. J. Inorg. Biochem..

[19] Graeme K.A., Pollack C.V. (1998). Heavy metal toxicity, part I: Arsenic and mercury. J. Emerg. Med..

[20] Hunter D., Russell D.S. (1954). Focal cerebral and cerebellar atrophy in a human subject due to organic mercury compounds. J. Neurol. Neurosurg. Psychiatry.

[21] O’shea J. (1990). ‘Two minutes with venus, two years with mercury’-mercury as an antisyphilitic chemotherapeutic agent. J. R. Soc. Med..

[22] Powell P.P. (1991). Minamata disease: A story of mercury’s malevolence. South Med. J..

[23] Obiri S., Dodoo D., Essumang D., Armah F. (2010). Cancer and non-cancer risk assessment from exposure to arsenic, copper, and cadmium in borehole, tap, and surface water in the Obuasi municipality, Ghana. Hum Ecol Risk Assess.

[24] Djedjibegovic J., Marjanovic A., Tahirovic D., Caklovica K., Turalic A., Lugusic A., Omeragic E., Sober M., Caklovica F. (2020). Heavy metals in commercial fish and seafood products and risk assessment in adult population in Bosnia and Herzegovina. Sci. Rep..

[25] Pandion K., Khalith S.M., Ravindran B., Chandrasekaran M., Rajagopal R., Alfarhan A., Chang S.W., Ayyamperumal R., Mukherjee A., Arunachalam K.D. (2022). Potential health risk caused by heavy metal associated with seafood consumption around coastal area. Environ. Pollut..

[26] Yabanli M., Tay S. (2021). Selenium and mercury balance in sea bream obtained from different living environments in Turkey: A risk assessment for the consumer health. Environ. Sci. Pollut. Res..

[27] Yabanli M., Tay S., Giannetto D. (2016). Human health risk assessment from arsenic exposure after sea bream (*Sparus aurata*) consumption in Aegean Region, Turkey. Bulg. J. Vet. Med..

[28] U.S. Environmental Protection Agency (USEPA) (1989). Risk Assessment Guidance for Superfund, Vol. 1. Human Health Evaluation Manual (Part A). EPA/540/1-89/02. Office of Emergency and Remedial Response.

[29] Hwang H.-J., Hwang G.-H., Ahn S.-M., Kim Y.-Y., Shin H.-S. (2022). Risk assessment and determination of heavy metals in home meal replacement products by using inductively coupled plasma mass spectrometry and direct mercury analyzer. Foods.

[30] de Souza-Araujo J., Hussey N.E., Hauser-Davis R.A., Rosa A.H., de Oliveira Lima M., Giarrizzo T. (2022). Human risk assessment of toxic elements (As, Cd, Hg, Pb) in marine fish from the Amazon. Chemosphere.

[31] Korea Disease Control and Prevention Agency (KDCA) (2020). Korea National Health and Nutrition Examination Survey.

[32] National Health Insurance Service (NHIS) (2022). National Health Check-Up Statistical Data.

[33] U.S. Environmental Protection Agency (USEPA) (2022). Integrated Risk Information System.

[34] Luo L., Wang B., Jiang J., Fitzgerald M., Huang Q., Yu Z., Li H., Zhang J., Wei J., Yang C. (2021). Heavy metal contaminations in herbal medicines: Determination, comprehensive risk assessments, and solutions. Front. Pharmacol..

[35] Kopru S., Cadir M., Soylak M. (2022). Investigation of trace elements in vegan foods by ICP-MS after microwave digestion. Biol. Trace Elem. Res..

[36] Habte G., Choi J.Y., Nho E.Y., Oh S.Y., Khan N., Choi H., Park K.S., Kim K.S. (2015). Determination of toxic heavy metal levels in commonly consumed species of shrimp and shellfish using ICP-MS/OES. Food Sci. Biotechnol..

[37] Döker S., Uslu M. (2014). Aerosol dilution technique for direct determination of ultra-trace levels of Cr, Mn, Fe, Co, Ni, Cu, and Zn in edible salt samples by collision/reaction cell inductively coupled plasma mass spectrometry (CRC-ICP-MS). Food Anal. Methods.

[38] AOAC (2012). AOAC International Methods Committee Guidelines for Validation of Qualitative and Quantitative Food Microbiological Official Methods of Analysis.

[39] Ministry of Food and Drug Safety (MFDS) (2022). Food Standards and Specification.

[40] EU (2006). Commission Regulation (EC) No 1881/2006 of 19 December 2006 Setting Maximum Levels for Certain Contaminants in Foodstuffs.

[41] CODEX General Standard for Contaminants and Toxins in Food and Feed. https://www.fao.org/fao-who-codexalimentarius/sh-proxy/en/?lnk=1&url=https%253A%252F%252Fworkspace.fao.org%252Fsites%252Fcodex%252FStandards%252FCXS%2B193-1995%252FCXS_193e.pdf,.

[42] China Food & Drug Administration (CFDA) (2022). GB 2762-2022 National Food Safety Standard—Maximum Levels of Contaminants in Foods.

[43] Kim H.-Y., Kim J.-I., Kim J.-C., Park J.-E., Lee K.-J., Kim S.-I., Oh J.-H., Jang Y.-M. (2009). Survey of heavy metal contents of circulating agricultural products in Korea. Korean J. Food Sci. Technol..

[44] Masuda H. (2018). Arsenic cycling in the Earth’s crust and hydrosphere: Interaction between naturally occurring arsenic and human activities. Prog. Earth Planet. Sci..

[45] Hwang I.M., Lee H.M., Lee H.-W., Jung J.-H., Moon E.W., Khan N., Kim S.H. (2021). Determination of toxic elements and arsenic species in salted foods and sea salt by ICP–MS and HPLC–ICP–MS. ACS Omega.

[46] Craig P.J., Jenkins R. (2004). Organometallic compounds in the environment: An overview. Organic Metal and Metalloid Species in the Environment: Analysis, Distribution, Processes and Toxicological Evaluation.

[47] Mason R.P., Reinfelder J.R., Morel F.M. (1995). Bioaccumulation of mercury and methylmercury. Water Air Soil Pollut..

[48] Jensen S., Jernelöv A. (1969). Biological methylation of mercury in aquatic organisms. Nature.

[49] Agah H., Leermakers M., Elskens M., Fatemi S.M.R., Baeyens W. (2007). Total mercury and methyl mercury concentrations in fish from the Persian Gulf and the Caspian Sea. Water Air Soil Pollut..

[50] Yamashita Y., Omura Y., Okazaki E. (2005). Total mercury and methylmercury levels in commercially important fishes in Japan. Fish. Sci..

[51] Fernández B., Campillo J., Martínez-Gómez C., Benedicto J. (2010). Antioxidant responses in gills of mussel (*Mytilus galloprovincialis*) as biomarkers of environmental stress along the Spanish Mediterranean coast. Aquat. Toxicol..

[52] Kamimura S. (1980). Influence of Copper and Zinc in Food Substance on the Accumulation of Cultured Oysters. Bull. Jpn. Soc. Sci. Fish..

[53] Phillips D.J. (1977). The use of biological indicator organisms to monitor trace metal pollution in marine and estuarine environments—A review. Environ. Pollut..

[54] Bustamante P., Grigioni S., Boucher-Rodoni R., Caurant F., Miramand P. (2000). Bioaccumulation of 12 trace elements in the tissues of the nautilus Nautilus macromphalus from New Caledonia. Mar. Pollut. Bull..

[55] Je J.G. (1990). Preliminary study on the cephalopod molluscs of the Korean waters. Rep. Korea Ocean. Res. Dev. Inst..

[56] Kim K.H., Kim Y.J., Heu M.S., Kim J.-S. (2016). Contamination and risk assessment of lead and cadmium in commonly consumed fishes as affected by habitat. Korean J. Fish Aquat. Sci..

[57] Liu Q., Liao Y., Shou L. (2018). Concentration and potential health risk of heavy metals in seafoods collected from Sanmen Bay and its adjacent areas, China. Mar. Pollut. Bull..

[58] Sunlu U. (2006). Trace metal levels in mussels (*Mytilus galloprovincialis* L. 1758) from Turkish Aegean Sea coast. Environ. Monit. Assess..

[59] Almela C., Algora S., Benito V., Clemente M., Devesa V., Suner M., Velez D., Montoro R. (2002). Heavy metal, total arsenic, and inorganic arsenic contents of algae food products. J. Agric. Food Chem..

[60] Hajrić D., Smajlović M., Antunović B., Smajlović A., Alagić D., Tahirović D., Brenjo D., Članjak-Kudra E., Djedjibegović J., Porobić A. (2022). Risk assessment of heavy metal exposure via consumption of fish and fish products from the retail market in Bosnia and Herzegovina. Food Control.

[61] Bonsignore M., Manta D.S., Mirto S., Quinci E.M., Ape F., Montalto V., Gristina M., Traina A., Sprovieri M. (2018). Bioaccumulation of heavy metals in fish, crustaceans, molluscs and echinoderms from the Tuscany coast. Ecotoxicol. Environ. Saf..

[62] Makedonski L., Peycheva K., Stancheva M. (2017). Determination of heavy metals in selected black sea fish species. Food Control.

[63] Leatherland T., Burton J., Culkin F., McCartney M., Morris R. (1973). Concentrations of some trace metals in pelagic organisms and of mercury in Northeast Atlantic Ocean water. Deep Sea Res. Oceanogr. Abstr..

[64] Monteiro L., Costa V., Furness R., Santos R. (1996). Mercury concentrations in prey fish indicate enhanced bioaccumulation in mesopelagic environments. Mar. Ecol. Prog. Ser..

[65] Velez C., Figueira E., Soares A., Freitas R. (2015). Spatial distribution and bioaccumulation patterns in three clam populations from a low contaminated ecosystem. Estuar. Coast. Shelf Sci..

[66] Liu J., Cao L., Dou S. (2017). Bioaccumulation of heavy metals and health risk assessment in three benthic bivalves along the coast of Laizhou Bay, China. Mar. Pollut. Bull..

[67] The Joint FAO/WHO Expert Committee on Food Additives (JECFA) (2022). Evaluation of Certain Food Additives and Contaminants: Ninety Third Report of the Joint FAO/WHO Expert Committee on Food.

[68] Shaheen N., Irfan N.M., Khan I.N., Islam S., Islam M.S., Ahmed M.K. (2016). Presence of heavy metals in fruits and vegetables: Health risk implications in Bangladesh. Chemosphere.

[69] Cho I.-S., Kim S.-J., Park A.-S., Kim J.-A., Jang J.-I., Lee S.-D., Yu I.-S., Shin Y.-S. (2020). The Content and Risk Assessment of Heavy Metals in Herbal Medicines used for Food and Drug. J. Food Hyg. Saf..

[70] Hughes M.F. (2002). Arsenic toxicity and potential mechanisms of action. Toxicol. Lett..

